# New insights into the actions of bisphosphonate zoledronic acid in breast cancer cells by dual RhoA-dependent and -independent effects

**DOI:** 10.1038/sj.bjc.6600925

**Published:** 2003-05-13

**Authors:** C Denoyelle, L Hong, J-P Vannier, J Soria, C Soria

**Affiliations:** 1 Laboratoire DIFEMA, Groupe de Recherche MERCI, UFR de Medecine et de Pharmacie, 76183 Rouen, France; 2 Laboratoire de Biochimie Sainte Marie, Hôtel Dieu, 75004 Paris, France; 3 INSERM U.553, Hôpital St Louis, 75010 Paris, France

**Keywords:** bisphosphonate, metastatic breast cancer, invasion, RhoA, SDF-1, CXCR-4, Cox-2

## Abstract

Zoledronic acid (ZOL) is a nitrogen-containing bisphosphonate and its use in reducing osteoporosis and cancer-induced osteolysis is increasing. Recent findings indicated that ZOL has a direct effect on cancer cells. In this study, the effect of ZOL was examined on the aggressive MDA-MB-231 breast cancer cell line. ZOL induces an important inhibition of cell invasion at low concentrations (1 *μ*M). This is not explained by modifications of proteases involved in cell invasiveness (matrix metalloproteinases and urokinase-type plasminogen activator), but by a disorganisation of actin cytoskeleton due to RhoA inhibition related to its defective prenylation as it was reversed by geranylgeraniol (GGOH) and mimicked by the Rho selective inhibitor C3 exoenzyme. In addition, ZOL inhibits the chemotactic effect induced by stromal cell-derived factor 1(SDF-1), a chemokine greatly involved in cancer metastasis to bone. This effect is related to both reduction of cell motility induced by RhoA inhibition and to a decreased expression of CXCR-4, the SDF-1 receptor. Finally, ZOL reduces Cox-2 expression and, consequently, the secretion of prostaglandins E2 (PGE2) in a RhoA-independent manner. This inhibition could contribute to bone protection in breast cancers because PGE2 stimulates osteoclast-mediated bone resorption. In summary, new insights in the mechanism of ZOL action on aggressive breast cancer cells are demonstrated and could explain its beneficial action in both the reduction of osteolysis and prevention of metastasis.

The main cause of treatment failure for cancer patients is metastasis, a multistep and complex process leading to the formation of secondary tumours from the original cancer. Breast cancers preferentially metastasise into the skeleton, causing specific clinical complications: hypercalcaemia, bone fractures and pain (Paget, 1889; Mundy, 1997). Bone metastases in breast cancer account significantly for considerable morbidity and mortality. Indeed, 80% of patients with metastatic breast cancer will develop bone metastases and survive for approximately 2 years (Coleman and Rubens, 1987).

Bisphosphonates (BPs) have proven useful in the treatment of bone metastases in breast cancer patients and have improved their quality of life in a number of clinical trials (Theriault *et al*, 1999; Lipton *et al*, 2000). In the first years of BP use, the efficacy of these substances was thought to lie entirely in the antiproliferative and apoptotic effects on osteoclasts (Hughes *et al*, 1995; Selander *et al*, 1996). However, it is now suggested that these favourable effects may involve not only the inhibition of osteoclast-mediated bone resorption but also a direct antitumour effect on cancer cells (reviewed by Senaratne and Colston, 2002). Additionally, BPs have been reported to prevent attachment and spreading of breast and prostate cancer cells onto bone matrices (Boissier *et al*, 1997). A recent study using an *in vitro* model of cell invasion has also suggested that BPs may inhibit the early event in the formation of bone metastases (Boissier *et al*, 2000). Furthermore, several investigations have reported the inhibition of cell growth and cell survival by BPs on breast cancer cells (Fromigue *et al*, 2000; Senaratne *et al*, 2000; Hiraga *et al*, 2001; Jagdev *et al*, 2001).

While all these data support a direct action on tumour cells *in vitro* and is animal models, the molecular mechanism(s) of BP action remain(s) unclear. Two classes of BPs have been described. The first generation consists in non-nitrogen-containing BPs that are metabolised in nonhydrolysable ATP analogues, whereas the more recent generations, including zoledronic acid (ZOL), are nitrogen-containing BPs (N-BPs). One possible mechanism by which N-BPs mediate their effects is based on their ability to inhibit some of the enzymes involved in the mevalonate (MVA) pathway, such as farnesylpyrophosphate synthase (van Beek *et al*, 1999; Bergstrom *et al*, 2000) and/or geranylgeranylpyrophosphate synthase (Coxon *et al*, 2000), thus blocking the generation of isoprenoid compounds, farnesyl pyrophosphate (FPP) and/ or geranylgeranyl pyrophosphate (GGPP), respectively. These intermediates are required for post-translational prenylation (farnesylation and geranylgeranylation) of key regulatory proteins, a step that is needed to their attachment to the plasma membrane, where they are fully active and can exert their biological function (Sinensky, 2000). Consequently, the identification of the proteins that became unprenylated following N-BP treatment should be of great interest in understanding the anticancer action of N-BPs.

Among all the potential candidate's proteins, the small GTPases of Ras and Rho families could be attractive targets for two reasons. First, their prenylation is a functional requirement: farnesylation of Ras and geranylgeranylation of Rho proteins occur, respectively, through the transfer of a 15-carbon farnesyl isoprenoid from FPP by the action of farnesyltransferase enzyme (FTase) and one or two 20-carbon geranylgeranyl isoprenoids from GGPP by the action of geranylgeranyltransferase enzyme (GGTase). Second, the small GTPases of Ras and Rho families are widely involved in human tumorigenesis and metastasis, either through constitutive activation caused by mutations or by overexpression (Adjei, 2001; Sahai and Marshall, 2002). Then, rendering insensitive to regulatory signals, they lead to uncontrolled cell proliferation, inhibition of apoptosis and enhanced angiogenesis, all main aspects of tumour development. Furthermore, Ras and Rho proteins are also involved in carcinoma cell motility (reviewed by Oxford and Theodorescu, 2003). For example, activated Ras was shown to stimulate migration of breast cancer cells (Keely *et al*, 1999). Additionally, the regulation of the actin cytoskeleton by Rho GTPases has been implicated in promoting a variety of cellular processes such as changes in morphology, motility and adhesion that contribute to invasion and metastasis of cancer cells in different models, either *in vitro* or *in vivo* (Jaffe and Hall, 2002). In colon carcinoma cells, expression of a dominant-negative RhoA resulted in the attenuation of cell invasion stimulated by the integrin *α*6*β*4 (O'Connor *et al*, 2000). In the same way, cells transformed by the activated *RhoA* gene (del Peso *et al*, 1997) or cells expressing a constitutively active form of RhoA (Yoshioka *et al*, 1998) have greatly promoted invasive ability, contributing to the acquisition of a metastatic phenotype *in vivo.* Finally, we recently demonstrated that RhoA activation contributes to breast cancer cell aggressivity (Denoyelle *et al*, 2003). Therefore, it would be interesting to determine whether the anticancer action of N-BPs could be related to the inhibition of Ras and/or RhoA prenylation, following the decrease of FPP and/or GGPP synthesis. Recently, a part of the enigma was resolved by a study demonstrating that ZOL-mediated apoptosis in breast cancer cells may be initiated by inhibition of Ras prenylation (Senaratne *et al*, 2002). However, apoptosis of these cancer cells occurs only at high concentrations (100 *μ*M), higher than pharmacological concentrations. Although one study suggested that the concentration of BP could reach as high a value as 800 *μ*M at the osteoclast–bone interface (Sato *et al*, 1991), others reported that an estimated concentration of BP is about 1 *μ*M in the metastatic tumour nest in bone (Usui *et al*, 1997). Thus, it is uncertain whether this proposed mechanism of action is also relevant for the clinical effect of N-BPs. In contrast, only very low concentrations of ZOL (10^−12^–10^−6^ M) are able to inhibit cancer cell invasion (Boissier *et al*, 2000). Recently, it was proposed that this inhibitory effect was mediated by the inhibition of the mevalonate pathway in a prostate cancer cell line, as it was reversed by geranylgeraniol (GGOH) and farnesol (FOH) (Virtanen *et al*, 2002). In the present study, we thus attempted to analyse whether the impairment of Ras and/or RhoA prenylation could be a potential mechanism by which ZOL mediated its anti-invasive effect on the aggressive MDA-MB-231 breast cancer cell line. In addition, matrix metalloproteinases (MMPs) and urokinase-type plasminogen activator (u-PA)/u-PA receptor (u-PAR) contribute to cancer cell invasion (Blasi, 1999). It was already demonstrated that the inhibition of MDA-MB-231 cell invasion is not explained by a decrease in MMP secretion as it was observed for concentrations higher than required for cell invasion inhibition (Boissier *et al*, 2000). Then, the effect of ZOL was examined on u-PA/u-PAR expression in MDA-MB-231 cells.

Next, based on the observation that BPs reduce the release of bone-derived growth factors and cytokines associated with bone resorption, which have the potential to attract cancer cells to bone, it is possible that BP therapy may prevent the development of bone metastases. Two clinical trials of patients with breast cancer demonstrated that BPs, given in adjuvant setting, reduce the incidence of skeletal metastasis with a consequent improvement in survival (Diel *et al*, 1998; Powles *et al*, 2002). However, other authors reported opposite results (Saarto *et al*, 2001). Recently, it was demonstrated that cancer cells use the stromal cell-derived factor 1 (SDF-1)/chemokine receptor of SDF-1 (CXCR-4) pathway to spread to bone (Taichman *et al*, 2002). CXCR-4 is greatly expressed on malignant breast cancer cells in comparison to normal epithelial cells (Müller *et al*, 2001). In addition, a high level of SDF-1 was noted in bone as well as in all target organs for breast-cancer metastasis and, in *Nude* mice, a neutralising anti-CXCR-4 antibody induces a significant inhibition of breast-cancer metastasis *in vivo*, indicating a major role of the SDF-1/CXCR-4 pathway in the metastatic process. Therefore, we evaluated the effect of ZOL on CXCR-4 expression and on the chemotactic effect induced by SDF-1.

Finally, it is well established that a ‘vicious cycle’ between cancer cells and osteoclasts favours cancer-induced osteolysis (Okada *et al*, 2000). It was demonstrated that invasive mammary cell lines, which constitutively express inducible Cox-2, enhance osteoclast formation through the production of high levels of prostaglandins E2 (PGE2) and subsequently induce an increase of osteolysis (Ono *et al*, 2002). We thus examined whether ZOL could affect the constitutive expression of Cox-2 in MDA-MB-231 cells.

## MATERIALS AND METHODS

### Cell culture

A human breast carcinoma cell line MDA-MB-231 was used in this study. MDA-MB-231 cells were grown in RPMI 1640 medium (Eurobio, Les Ulis, France), 10% foetal calf serum (FCS, Costar, Brumath, France), 2 mM L-glutamine (Gibco BRL, New York, NY, USA), 100 IU ml^−1^ penicillin (Sarbach, Suresnes, France) and 100 *μ*g ml^−1^ streptomycin (Diamant, Puteaux, France). Cells were cultured at 37°C in a humidified 5% CO_2_ atmosphere. Then, adherent cells were incubated with indicated concentrations of ZOL (Zometa®, Novartis) during different periods. The role of MVA pathway enzymes was studied by treating cells with 100 *μ*M MVA, 10 *μ*M FOH (analogue of FPP), 10 *μ*M GGOH (analogue of GGPP), or 10 *μ*M squalene (SQUA) (Sigma, Saint Quentin Fallavier, France). The farnesyltransferase inhibitor FTI-277, the geranylgeranyltransferase inhibitor GGTI-298 and the C3 exoenzyme from *Clostridium botulinum* C3 transferase (C3 Exo), a specific inhibitor of RhoA, were purchased from Calbiochem (San Diego, CA, USA).

### Cell proliferation

For the proliferation assay, we used the minimal concentration of FCS (2%) to allow sufficient viability of MDA-MB-231 cells. Briefly after trypsinisation, the cells were seeded at a concentration of 5 × 10^4^ cells per well in a 24-well plate (Costar, Cambridge, MA, USA) and incubated with ZOL. Cell number was measured on day 3 for MDA-MB-231 with a particle counter (Coulter Z1, Coultronics, France) after detachment with a nonenzymatic cell dissociation solution (Sigma).

### Flow cytometry analysis

Flow cytometry analysis was performed as previously described (Denoyelle *et al*, 2001). Tumour cells were detached by a nonenzymatic cell dissociation solution and washed twice in cold PBS. The phosphatidylserine expression on the surface of breast-cancer cells was determined using an Annexin V commercial kit (Immunotech, Marseille, France). Briefly, cells were incubated with 10 *μ*l of a fluorescein–isothiocyanate (FITC)-conjugated Annexin V. Staining with propidium iodide (0.3 *μ*g ml^−1^) was performed to confirm the absence of cell membrane permeability. u-PAR expression on MDA-MB-231 cells was determined by direct immunofluorescence, while u-PA and CXCR-4 were detected by indirect immunofluorescence. Approximately 5 × 10^5^ cells were incubated for 15 min at 4°C with 5 *μ*l of specific antibodies (1 mg ml^−1^). After two washes, the cell suspension was immediately analysed in a flow cytometer (EPICS XL-MCL, Coulter, USA), when antibody was directly conjugated to phycoerythrin (u-PAR, Immunotech), while another 15 min incubation with an FITC-labelled F(ab′)2 fraction of goat anti-mouse IgG1 (10 *μ*g ml^−1^, Immunotech) was carried out for the detection of u-PA (American Diagnostic, Greenwich, CT, USA) and CXCR-4 (R&D Systems, Abingdon, UK) antibodies. Data are expressed as the percentage of fluorescent cells or as the specific mean channel fluorescence intensity (MFI). Specific MFI was calculated for each sample by subtracting the background MFI produced by an irrelevant antibody from the MFI generated by the specific antibody.

### Invasion assay on a Matrigel-coated membrane in a Transwell

An 8-*μ*m-diameter Pore Transwell (Dutscher, Brumath, France) were coated with 500 *μ*l of Matrigel (Becton Dickinson Europe, Meylan, France) diluted at 100 *μ*g ml^−1^. Breast tumour cells were detached by the nonenzymatic cell dissociation solution, washed twice with PBS, and 2 × 10^5^ cells in RPMI 1640 with 0.2 mg ml^−1^ bovine serum albumin (BSA, Sigma) were seeded in the upper chamber of the Matrigel-coated insert. The lower chamber was filled with 1 ml of RPMI 1640 together with 2 mg ml^−1^ BSA and basic fibroblast growth factor (bFGF, 20 ng ml^−1^) (R&D Systems) to induce invasion. The chemotactic effect induced by SDF-1 was studied by realising a gradient, which was achieved by adding SDF-1 (200 ng ml^−1^) in the lower compartment. After 18 h of incubation, the nonmigrating cells in the upper chamber were gently scraped, and the adherent cells present on the lower surface of the insert were stained by May–Grünewald–Giemsa and counted by light microscopy, 10 fields (magnification × 200) were counted for each insert. To verify that observed responses are dependent on CXCR-4 receptor binding, the cells were incubated for 30 min at 4°C with 100 *μ*g of the CXCR-4 antibody before being seeded in the upper chamber.

### Separation of particulate and cytosolic fractions

After different incubation times with ZOL, separation of particulate and cytosolic fractions was performed as previously described (Denoyelle *et al*, 2003). Briefly, MDA-MB-231 cells were washed with cold PBS, lysed in ice-cold buffer that contained phosphatase and protease inhibitors and centrifuged at 100 000 **g** for 30 min at 4°C. The supernatant was collected as the cytosolic fraction. Pellets were homogenised in the above-mentioned lysis buffer containing 2% Triton X-114 (Sigma) and centrifuged at 800 **g** for 10 min at 4°C. The supernatant was collected and was referred to as the membrane fraction. The protein concentration was determined according to the method of Bradford using the Bio-Rad protein assay (Hercules, CA, USA).

### Confocal microscopy analysis of actin cytoskeleton on MDA-MB-231 cells

MDA-MB-231 was cultured in four-well Glass Lab-Tek chamber slides (Nunc, Roskilde, Denmark). The confocal microscopy analysis of actin filaments was performed after an 18 h incubation with 1 *μ*M ZOL as previously described (Denoyelle *et al*, 2001). Actin filaments were visualised by phalloidin–tetramethylrhodamine–isothiocyanate (TRITC) conjugate (Sigma). Computer-assisted image analysis of fluorescence was performed using a confocal microscopy scanning laser microscope (Leica TCS, wavelength excitation 488 nm, emission 580 nm).

### Western blotting

Equal amounts of protein extracts (50 *μ*g) were subjected to PAGE (15% for RhoA, Ras and 10% for Cox-2) under denaturing conditions (SDS–PAGE). Proteins were electrotransferred onto polyvinylidene difluoride (PVDF) membrane (Amersham Pharmacia Biotech, Saclay, France), as described (Denoyelle *et al*, 2001). Membranes were immunoblotted overnight with RhoA or pan-Ras polyclonal antibody (1 : 500, from Santa Cruz, SA, USA) or Cox-2 monoclonal antibody (1 : 250, from Transduction laboratories), followed by incubation with the appropriate horseradish peroxidase-conjugated secondary antibody (1 : 10 000, Dako, Trappes, France), then developed with enhanced chemoluminescence reagent (ECL) solution (Amersham Pharmacia Biotech) and exposed to Kodak X-OMAT films. For reprobing, membranes were stripped with a solution containing 50 mM Tris-HCl (pH 6.8), 2% SDS and 100 mM 2-mercaptoethanol for 30 min at 50°C. Blots were rehybridised with *β*-actin polyclonal antibody (1 : 5000, Sigma) to control protein loading. Each immunoblot is representative of three distinct experiments.

### Total RNA extraction and RT–PCR

After the indicated incubation time of MDA-MB-231 cells with 1 *μ*M ZOL, cells were detached and washed twice in PBS. Total RNA extraction was performed using the SV Total RNA Isolation system (Promega, Madison, WI, USA) according to the manufacturer's instructions. Primers were chosen using biomolecular sequence databases (Genbank) and oligonucleotides were synthesised by ProOligos (Paris, France); the sequences were as follows: *cox-2* (forward primer, 5′-cggacaggattctatggaga-3′; reverse primer, 5′-caatcatcaggcacaggagg-3′), *GAPDH* (forward primer, 5′-cggagtcaacggatttggtcgtat-3′; reverse primer, 5′-cgctcctggaagatggtgatgg-3′). RT–PCR was carried out using the ‘Access RT–PCR system’ (Promega) according to the manufacturer's instructions. After PCR, 15 *μ*l of products and standard DNA ladder were run on a 1.5% agarose gel stained with ethidium bromide. The predicted sizes for cox-2 and GAPDH PCR products were, respectively, 300 and 222 bp.

### ELISA assay

The measurement of PGE2 was performed using an ELISA assay, according to the manufacturer's instructions (R&D Systems).

### Statistical analysis

Statistical significance was determined by the Student's *t*-test using the InStat Software (Sigma).

## RESULTS

### Apoptosis and inhibition of MDA-MB-231 cell proliferation requires high concentrations of ZOL

**Figure 1:**
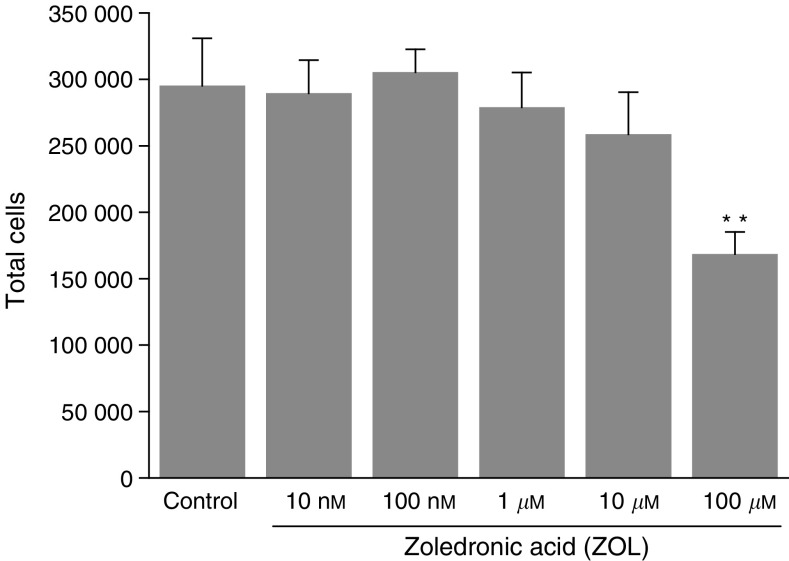
Effect of zoledronic acid (ZOL) on MDA-MB-231 cell proliferation. Cells (5 × 10^5^) were seeded per well and cells were counted in a particle counter after 3 days of culture with a minimal concentration of FCS (2%) to ensure viability of the cells in the presence or absence of indicated concentrations of ZOL. Results are mean±s.e.m. of four independent experiments (^**^P<0.01).

The effects of various concentrations of ZOL on the growth rates of MDA-MB-231 cells were studied by measuring the cell number in the absence or presence of ZOL at different concentrations after a 3-day incubation. Furthermore, the effects of ZOL on apoptosis were also assessed by flow cytometry using Annexin V conjugated to fluorescein, an early indicator of apoptosis. Positive control was done using Taxol (a well-known mitotic spindle toxin). ZOL had no effects on MDA-MB-231 cell proliferation at low concentrations (<10 *μ*M) (Figure 1

). In contrast, cell numbers were significantly reduced at a concentration of 100 *μ*M ZOL. Likewise, ZOL did not induce apoptosis on this cell line for low concentrations, whereas Annexin V binding was detected at the tumour cell surface when they were treated with 100 *μ*M ZOL (Table 1

**Table 1:** Zoledronic acid (ZOL) induces apoptosis in MDA-MB-231 cells only at high concentrations

		**ZOL (*μ*M)**					
	**Control**	**0.01**	**0.1**	**1**	**10**	**100**	**Taxol**
Annexin V	7.9±1.7	12.6±2.3	8.2±2.8	9.9±3.2	13.7±2.6	35.2*±5.3	64.2**±4.8
Propidium iodide	4.2±1.8	6.7±2.3	5.9±3.1	5.6±2.9	6.5±1.2	5.1±1.9	6.9±2.5

Confluent MDA-MB-231 cells were incubated for 18 h with ZOL at indicated concentrations. Taxol was used as positive control. Results are the mean±s.e.m. of three independent experiments and are expressed as the percentage of positive cells (Bold indicates significance;

* *P*<0.05,

** *P*<0.01).

).

### Inhibition of MDA-MB-231 cell invasion through Matrigel by low concentrations of ZOL

**Figure 2:**
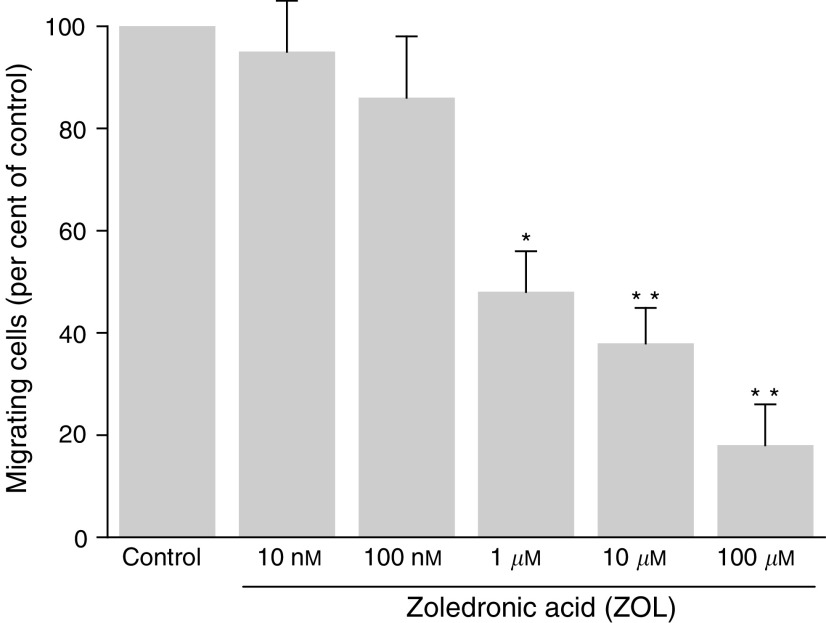
Effect of increasing concentrations of zoledronic acid (ZOL) on MDA-MB-231 cell invasiveness through Matrigel. MDA-MB-231 cells were treated for 18 h by indicated concentrations of ZOL. After detachment and two washes in PBS, 2 × 10^5^ cells were added in the upper Transwell chamber coated with Matrigel, as described in the Materials and Methods section. After 18 h incubation at 37°C, the cells in the upper part of the invasion chamber were gently detached and cells that had traversed the filter were counted by light microscopy after May–Grünewald–Giemsa coloration. In all, 10 fields (magnification × 200) were counted for each insert. Data are expressed as the percentage (as compared to control) of the mean±s.e.m. of five independent experiments. (^*^P<0.05, ^**^P<0.01).

Under our conditions, as shown in Figure 2

, MDA-MB-231 cells were highly invasive in Matrigel-coated chambers. Treatment of MDA-MB-231 cells for 18 h with ZOL at low concentrations (from 10^−7^ M) inhibited MDA-MB-231 cell invasion in the *in vitro* invasion assay in a dose-dependent manner (Figure 2). The number of invading cells was decreased by 62.1±3.8% (*P*<0.01) following treatment with 1 *μ*M ZOL. Consequently, a 1 *μ*M concentration of ZOL was chosen for treatment of MDA-MB-231 cells for the following experiments.

### Inhibition of MDA-MB-231 cell invasion by ZOL is reversed by GGOH

**Figure 3:**
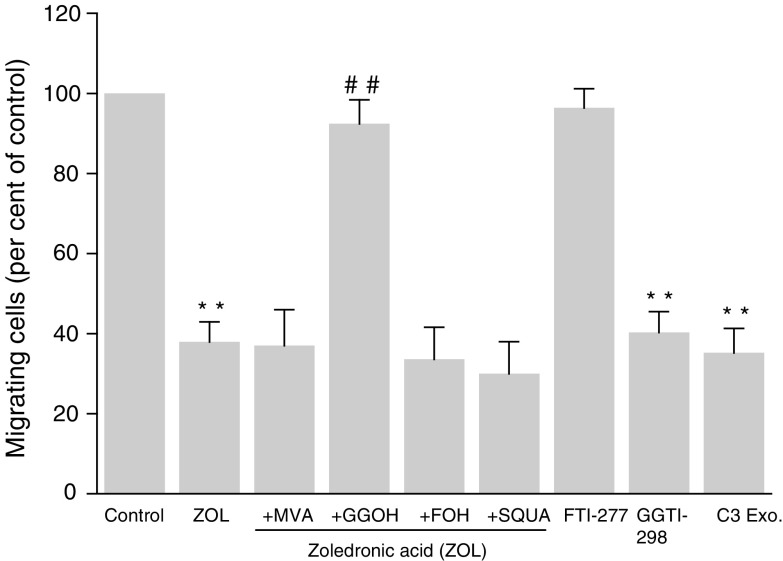
Effect of the mevalonate (MVA) pathway metabolites on zoledronic acid (ZOL)-induced MDA-MB-231 cell invasion inhibition – Comparison with the effect of FTase inhibitor (FTI-277), GGTase inhibitor (GGTI-298) and C3 exoenzyme. MDA-MB-231 cells were treated for 18 h with 5 *μ*g ml^−1^ C3 exoenzyme or 10 *μ*M FTI-277 or 10 *μ*M GGTI-298 or 1 *μ*M ZOL in the presence or absence of different MVA pathway metabolites–100 *μ*M mevalonate (MVA), 10 *μ*M farnesol (FOH), 10 *μ*M geranylgeraniol (GGOH) and 10 *μ*M squalene (SQUA) and experiments were performed as indicated in Figure 2. Data are expressed as the percentage (as compared to control) of the mean±s.e.m. of five independent experiments. Significant difference from nontreatment control (^**^); from ZOL-treated cells (^##^) (^**,##^*P*<0.01).

It was reported that N-BPs inhibit enzymes of the MVA pathway, and thus the synthesis of isoprenoid intermediates. To determine whether the inhibition of isoprenoids by ZOL is responsible for its inhibitory effect on cell invasion, we tested whether the inhibition of cell invasion by ZOL was reversed by the MVA pathway metabolites (MVA, FOH, GGOH and SQUA). It was verified that the simple addition of these intermediates has no effects on the invasion of untreated MDA-MB-231 cells (data not shown). The invasion-suppressive effect of ZOL on MDA-MB-231 cells was circumvented by the addition of 10 *μ*M GGOH, which restores geranylgeranylation (the percentages of invading cells in comparison to untreated cells were, respectively, for 1 *μ*M ZOL-treated cells, 37.9±3.8 and 92.4±2.8% in the absence and presence of GGOH) (Figure 3

). In contrast, MVA, SQUA and FOH (which restores farnesylation) did not reverse the anti-invasive effect of ZOL. In addition, to confirm that the anti-invasive effect of ZOL is mediated by inhibiting protein geranylgeranylation, we next examined whether the GGTase I inhibitor, GGTI-298, mimicked the effect of ZOL. As shown in Figure 3, treatment of MDA-MB-231 cells with GGTI-298 caused a significant decrease of cell invasion, while FTI-277, an inhibitor of FTase, at the same concentrations, was devoid of effect. Therefore, these results suggest that inhibition of geranylgeranylated proteins (such as Rho) rather than farnesylated proteins (such as Ras) seems to be important to explain the anti-invasive action of ZOL. Since the small GTPase RhoA requires geranylgeranylation to promote cancer invasion, we suggested that RhoA could represent a potential candidate to mediate ZOL effects. In support of this hypothesis, it was found that the addition of *Clostridium botulinum* C3 transferase (C3 exoenzyme), a widely accepted RhoA inhibitor (Sekine *et al*, 1989), mimicked ZOL action on MDA-MB-231 cells.

### ZOL mediates MDA-MB-231 cell invasion inhibition by preventing RhoA translocation from the cytosol to cell membrane

**Figure 4:**
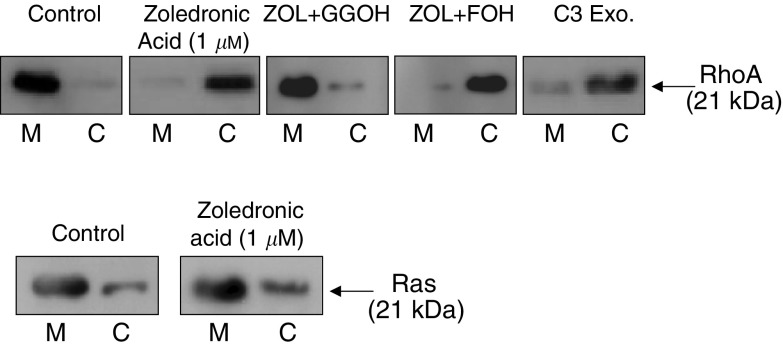
Zoledronic acid (ZOL) prevents the translocation of RhoA from cytoplasm to the cell membrane in MDA-MB-231 cells (**A**), but has no effect on Ras localisation (**B**). (A) MDA-MB-231 cells were treated with ZOL (1 *μ*M) in the presence or absence of GGOH (10 *μ*M) or FOH (10 *μ*M) for 18 h. C3 exoenzyme was used as positive control. (B) MDA-MB-231 cells were treated or not for 18 h with 1 *μ*M ZOL. Cells were then lysed and the membrane (M) and cytosolic (C) fractions were separated by ultracentrifugation. RhoA (A) and Ras (B) were detected by immunoblotting using RhoA and Ras polyclonal antibodies. These results are representative of three independent experiments.

Since the small GTPase RhoA must be targeted to the plasma membrane for its activation (Yoshioka *et al*, 1998), we examined the effect of ZOL on the translocation of RhoA protein from the cytosol to the membrane fraction in MDA-MB-231 cells. In control MDA-MB-231 cells, RhoA is predominantly associated with the cell membrane, suggesting that in these cancer cells the overexpression of RhoA facilitates its translocation from the cytosol to the cell membrane, as previously described (Yoshioka *et al*, 1998; Fritz *et al*, 1999). In contrast, after treatment with 1 *μ*M ZOL for 18 h, RhoA was almost found in the cytosol fraction and the amount of this protein associated with the cell membrane was greatly reduced (Figure 4A

). In addition, we observed that the inhibiting effect of ZOL on the RhoA membrane localisation was prevented by the addition of GGOH, but not FOH. C3 exoenzyme was used as positive control. Therefore, these results suggested that RhoA seems to be the main target of the inhibitory effect induced by ZOL on cell invasion. Additionally, because it was recently suggested that the treatment of MDA-MB-231 with high concentrations of ZOL inhibits generation of FPP leading to decreased prenylation of Ras (Senaratne *et al*, 2002), we also analysed the effect of ZOL on the cellular localisation of Ras. In control cells, Ras was predominantly associated with cell membrane fraction because MDA-MB-231 cells are characterised by a Ras mutation, leading to its constitutive activation. According to our results (see above), ZOL did not inhibit the translocation of Ras from cytoplasm to the cell membrane at low concentration (1 *μ*M) after 18 h of treatment (Figure 4B).

### ZOL induces a disorganisation of actin cytoskeleton and a loss of stress fibres formation in MDA-MB-231 cells

**Figure 5:**
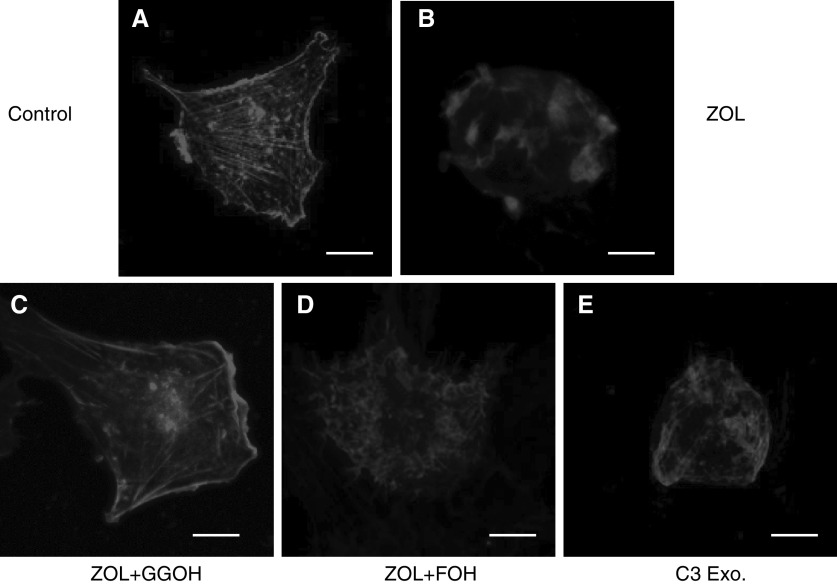
Morphological changes and reorganisation of actin cytoskeleton on MDA-MB-231 cells treated by zoledronic acid (ZOL). – Comparison with C3 exoenzyme. The actin cytoskeleton of MDA-MB-231 cells was labelled by phalloidin-TRITC and analysed by confocal microscopy. On untreated cells (**A**), note the actin stress fibres and cell spreading. Treatment for 18 h with 1 *μ*M ZOL (**B**) induced morphological changes of cells associated with disorganisation of actin stress fibres. The modifications induced by ZOL were reversed by geranylgeraniol (GGOH) (**C**) but not by farnesol (FOH) (**D**) and mimicked by C3 exoenzyme (**E**) (Scale bar=10 *μ*M).

Since RhoA is engaged in cytoskeleton reorganisation to promote cancer invasion, we studied the effect of ZOL on the morphology of MDA-MB-231 breast cancer cells. Untreated MDA-MB-231 cells were flat and well spread. In contrast, ZOL induced dramatic morphological changes characterised by a cell rounding and a disorganisation of actin cytoskeleton accompanied by a loss of stress fibres formation, which were clearly evidenced by confocal microscopy. Moreover, these morphological changes were rescued by the addition of GGOH, but not FOH (Figure 5

). Importantly, C3 exoenzyme mimicked the morphological changes induced by ZOL (Figure 5).

### ZOL inhibits the expression of u-PAR but not of u-PA in MDA-MB-231 cells

As u-PA associated with its receptor (u-PAR) is required to stimulate invasion of cancer cells (Blasi, 1999), we studied the effect of ZOL on the u-PA and u-PAR expressions by flow cytometry. After an 18 h incubation with 1 *μ*M ZOL, we observed a dose-dependent decrease of u-PAR antigen on the MDA-MB-231 cell surface (60% decrease) (Table 2

**Table 2:** Effect of zoledronic acid (ZOL) on u-PA and u-PAR expression on MDA-MB-231 cells

	**ZOL (*μ*M)**				
	**0.01**	**0.1**	**1**	**10**	**100**
u-PA	93.6±4.3	97.2±5.6	84.8±4.6	73.7±7.3	67.3*±5.8
					
u-PAR	96.6±5.1	64.2±9.4	43.6**±4.6	45.7*±7.1	39.3**±4.9

MDA-MB-231 cells were incubated for 18 h with increasing concentrations of ZOL and u-PA and its receptor u-PAR expressed at the membrane were analysed by flow cytometry. Data are expressed as the percentage (as compared to the controls) of the mean fluorescence intensity (MFI) of four separate experiments. (Bold indicates significance;

* *P*<0.05,

** *P*<0.01).

). In contrast, the inhibition of u-PA expression was only observed for 100 *μ*M ZOL, but not at lower concentrations (Table 2).

### Inhibition by ZOL of the chemotactic effect induced SDF-1 and CXCR-4 expression on MDA-MB-231 cells

**Figure 6:**
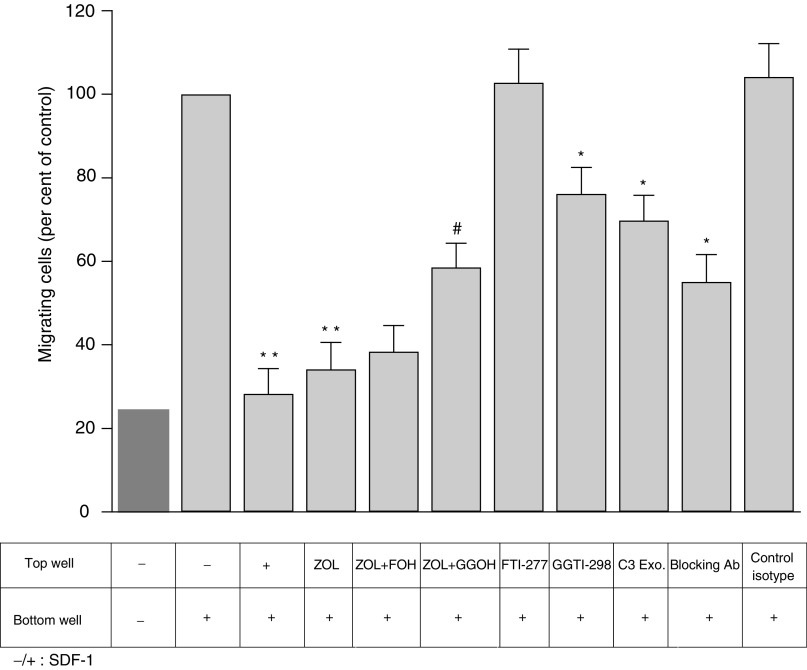
Zoledronic acid (ZOL) inhibits the chemotactic effect induced by SDF-1 on MDA-MB-231 cells – Partial reversion by geranylgeraniol (GGOH). Confluent MDA-MB-231 cells were treated for 18 h with 5 *μ*g ml^−1^ exoenzyme or 10 *μ*M FTI-277 or 10 *μ*M GGTI-298 or 1 *μ*M ZOL in the presence or absence of GGOH and farnesol (FOH) and seeded into the upper chamber of Transwell coated with Matrigel, and a gradient of SDF-1 (100 ng ml^−1^) was established by placing the chemokine in the lower chamber. Then the invasion was measured as described in Figure 2. The inhibitory effect of ZOL was compared with that of C3 exoenzyme and of neutralising antibody against CXCR-4. Results are expressed as the percentage (as compared to control) of the mean±s.e.m. of five independent experiments. Significant difference from nontreatment control (^*^); from ZOL-treated cells (^#^) (^*,#^*P*<0.05, ^**^P<0.01).

Since cancer cells use the SDF-1/CXCR-4 pathway to spread to bone (Taichman *et al*, 2002), we investigated whether ZOL may affect the SDF-1/CXCR-4 chemotaxis mechanism. Firstly, we verified that the addition of SDF-1 to the lower chamber induced invasion of MDA-MB-231 cells through a reconstituted basement membrane (Matrigel). The percentage of cells that invade the Matrigel was at least four times higher when SDF-1 was added in the lower chamber, indicating a potent chemotactic effect of SDF-1 on MDA-MB-231 cells (Figure 6

). As control, this SDF-1-mediated invasion could be abrogated by the addition of SDF-1 to both the upper and lower compartments of Boyden's chamber, confirming the specificity of the chemotactic response induced by this chemokine. Moreover, the addition of CXCR-4 neutralising antibody to the top of the culture chamber, but not an isotype control, decreased MDA-MB-231 cell invasion. This provides verification that the chemotactic effect induced by SDF-1 is mostly dependent on CXCR-4 receptor binding (Figure 6). Next, the ability of ZOL to influence SDF-1-induced breast carcinoma chemotactic effect was studied. The number of cells that invade Matrigel was roughly the same in untreated cells and cells treated with both SDF-1 and ZOL. Therefore, ZOL inhibited both invasion and SDF-1 chemotactic effect of breast-cancer cells. However, in contrast to cell invasion, the inhibition of chemotactic effect by ZOL was only rescued by 60% by GGOH and partially mimicked by GGTI-298. In addition, the reduction of SDF-1-induced invasion by C3 exoenzyme is much lower than that observed with ZOL-treated cells (30 *vs* 70%) (Figure 6). These results indicate that the decrease of cell motility induced by RhoA inhibition was not the only mechanism responsible for this inhibition. Recently, a strong cell-surface expression of CXCR-4, the SDF-1 receptor, was described on the aggressive MDA-MB-231 breast-cancer cell line (Müller *et al*, 2001). We confirmed these data on these cells (59.8% of positive cells) by flow cytometry (Table 3

**Table 3:** Effect of zoledronic acid (ZOL) on the expression of chemokine receptor CXCR-4 on MDA-MB-231 cells

		**ZOL (1 *μ*M)**		
**Control**	**ZOL (1 *μ*M)**	**+GGOH**	**+FOH**	**C3 Exo.**
59.8±3.1	15.7**±2.3	18.6±4.7	13.1±1.5	47.5±6.1

MDA-MB-231 cells were treated or not by 1 *μ*M ZOL with or without geranylgeraniol (GGOH) and farnesol (FOH). The effect of ZOL was compared with that of C3 exoenzyme (C3 Exo). The chemokine receptor of SDF-1, CXCR-4, was detected by flow cytometry by indirect immunofluorescence using an FITC-conjugated anti-mouse Ab. Results are the mean±s.e.m. of four independent experiments and are expressed as the percentage of positive cells (Bold indicates significance;

** *P*<0.01).

). Subsequently, the effect of ZOL was tested on CXCR-4 expression. As indicated in Table 3, an 18 h treatment with 1 *μ*M ZOL reduced the CXCR-4 expression from 59.8 to 15.7% of positive cells. This decrease of CXCR-4 expression by ZOL was not reversed by GGOH and FOH and was not induced by C3 exoenzyme.

### Inhibition of Cox-2 expression and PGE2 release by ZOL on MDA-MB-231 cells

**Figure 7:**
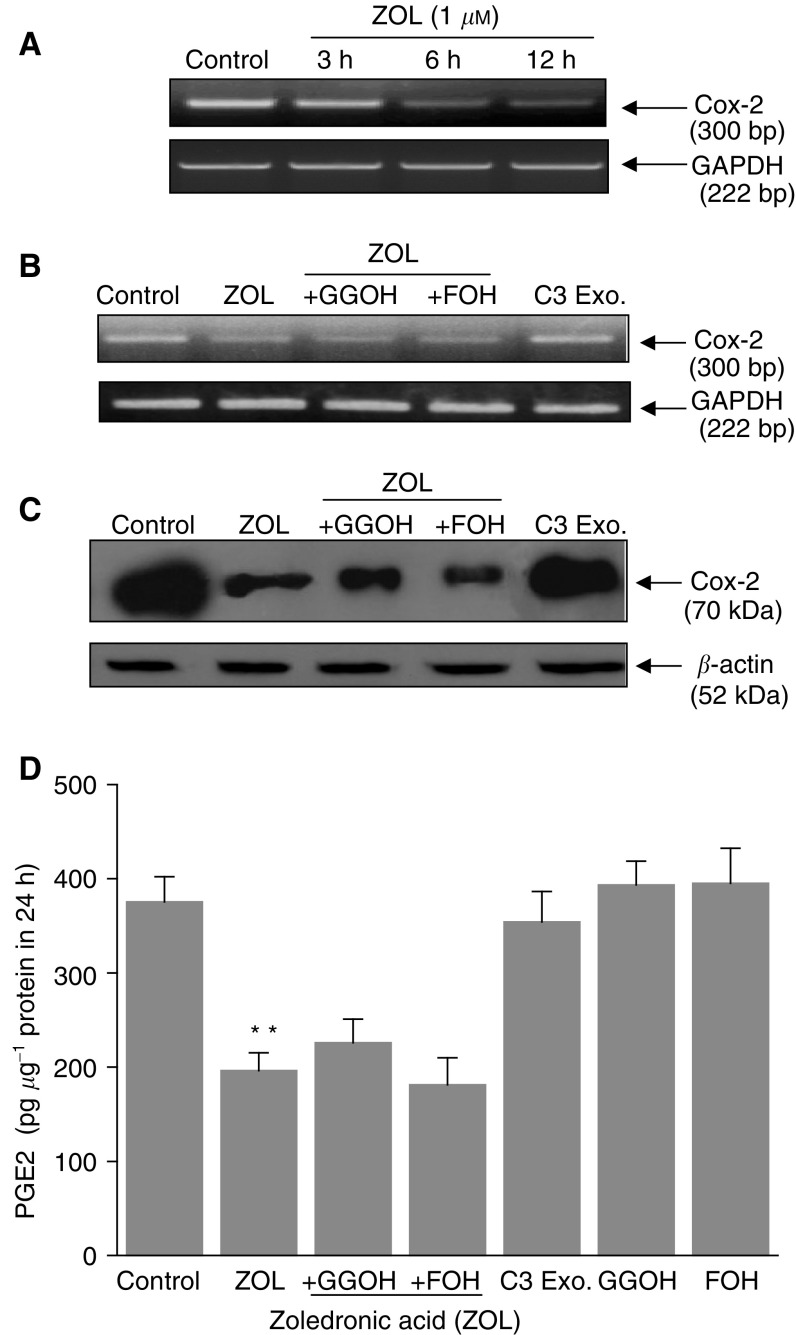
Effect of zoledronic acid (ZOL) on Cox-2 mRNA, protein levels and activity in MDA-MB-231 cells – Comparison with C3 exoenzyme. (**A**) Time course of ZOL on Cox-2 mRNA expression. MDA-MB-231 cells were treated for 3, 6 or 12 h with or without 1 *μ*M ZOL. RNA was extracted and analysed by RT–PCR. Sizes of PCR products for Cox-2 and GAPDH were, respectively, 300 and 222 bp. (**B**) Effect of ZOL on Cox-2 mRNA expression. MDA-MB-231 cells were incubated for 12 h with C3 exoenzyme (5 *μ*g ml^−1^) or with 1 *μ*M ZOL with or without geranylgeraniol (GGOH) (10 *μ*M) and farnesol (FOH) (10 *μ*M) and compared with untreated cells. RNA was extracted and analysed by RT–PCR. Sizes of PCR products for Cox-2 and GAPDH were, respectively, 300 and 222 bp. (**C**) Effect of ZOL on Cox-2 protein level. MDA-MB-231 cells were incubated with or without ZOL for 18 h and Cox-2 protein expression was examined by Western blot, using a monoclonal anti-Cox-2 antibody. Blots were developed with the enhanced chemoluminescence reagent (ECL). The membrane was also probed with *β*-actin to confirm equal loading. (**D**) Effect of ZOL on the secretion of PGE2 in the supernatant of MDA-MB-231 cells. The levels of PGE2 were measured by ELISA assay on supernatants of control and ZOL (1 *μ*M)-treated MDA-MB-231 cells in the presence or absence of GGOH and FOH. The effect of ZOL was compared with C3 exoenzyme. Results are mean±s.e.m. of three independent experiments and are expressed in picograms of PGE2 per microgram of protein (^**^P<0.01).

One mechanism that contributes to osteoclast activation and metastasis-induced osteolysis is the release of PGE2 by cancer cells (Ono *et al*, 2002). Therefore, we evaluated the action of ZOL on Cox-2 mRNA and protein expression in MDA-MB-231 cells. According to previous studies (Liu and Rose, 1996), MDA-MB-231 cells expressed a high constitutive level of inducible Cox-2, which was observed at both mRNA and protein levels. Interestingly, the Cox-2 transcript was greatly decreased from 6 h after 1 *μ*M ZOL treatment (Figure 7A

). In addition, when MDA-MB-231 cells were treated with 1 *μ*M ZOL for 18 h, a significant decrease of Cox-2 protein expression was noted (Figure 7B). As a consequence, it was found that treatment of cancer cells with ZOL induced a decrease in Cox-2 enzyme activity: a high level of PGE2 (375.4±26.7 pg *μ*g^−1^ protein in 24 h) was observed in the medium of untreated cells whereas exposure of cells to 1 *μ*M ZOL caused a clear decrease in PGE2 secretion (196.4±18.9 pg *μ*g^−1^ protein in 24 h, *P*<0.01) (Figure 7D). Importantly, the inhibition of Cox-2 expression by 1 *μ*M ZOL was not reversed by GGOH and FOH. This indicates that RhoA inhibition seems not to be involved in Cox-2 inhibition by ZOL. This RhoA-independent effect was also confirmed by the absence of the effect of C3 exoenzyme on Cox-2 at transcript and protein levels on these breast cancer cells (Figures 7B and C). This was also in good agreement with the observations that GGOH did not reverse the effect of ZOL on PGE2 secretion (225.8±38.6 pg *μ*g^−1^ protein in 24 h) and that C3 exoenzyme was also devoid of effect on PGE2 secretion.

## DISCUSSION

During the last years, many investigations have shown that BPs are targeted towards osteoclasts and protected against metastasis-induced osteolysis. However, BPs can also act directly on cancer cells. In the first part of this study, we analysed the action of ZOL, a third-generation N-BP that is increasingly being used in the treatment of bone metastases, on the proliferation and invasiveness of highly aggressive breast-cancer cells MDA-MB-231.

In agreement with recent studies already published (Fromigue *et al*, 2000; Hiraga *et al*, 2001; Jagdev *et al*, 2001), it was shown that ZOL inhibits MDA-MB-231 cell proliferation, but only for high concentrations (>100 *μ*M) which are certainly higher than the pharmacological concentrations obtained *in vivo*. However, such elevations of BP concentrations cannot be excluded, for short periods, within the bone metastases as the result of locally increased bone resorption in patients. The decrease of cell proliferation induced by ZOL could be related to the induction of apoptosis because it occurs in these cells at the same concentrations.

Next, the effect of ZOL was also studied on MDA-MB-231 cell invasion. After an 18 h incubation time, ZOL, at low concentrations (from 100 nM), displayed a potent anti-invasive property on MDA-MB-231 cells (62% decrease at 1 *μ*M) in the *in vitro* invasion assay through Matrigel. As ZOL did not induce apoptosis at these concentrations, the possibility that ZOL interfered with invasion by inducing cell death was excluded. This is also in agreement with the reported observations of Boissier *et al* (2000).

Additionally, we attempted to determine the mechanism involved in the ZOL-induced anti-invasive effect on MDA-MB-231 cells. This mechanism did not imply proteases involved in tumour invasion by inducing the degradation of the extracellular matrix (ECM). Indeed, neither MMP secretion nor u-PA expression was modified at concentrations that inhibit cell invasion. Only high concentrations were needed to reduce the secretion of both MMP-2 and MMP-9 (Boissier *et al*, 2000) and u-PA expression in MDA-MB-231 cells as shown in Table 2. In contrast, u-PAR expressed on the cell surface of MDA-MB-231 cells was dramatically reduced by ZOL at low concentrations (Table 2). u-PAR is a ligand for vitronectin, which is a common protein in mature bone microenvironment (Cooper *et al*, 2002). Consequently, the decrease of u-PAR by ZOL could contribute to the previously reported prevention of breast-cancer cell attachment onto bone matrices (van der Pluijm *et al*, 1996; Boissier *et al*, 1997).

Since the small GTPases of Ras and Rho families have to be prenylated to play an essential role in carcinoma cell invasion (Oxford and Theodorescu, 2003), we attempted to assess if the anti-invasive action of ZOL could be related to Ras and/or RhoA inactivation, following the decreased formation of FPP and GGPP, respectively. In this study, it was demonstrated that GGOH, which restores geranylgeranylation, but not FOH, which restores farnesylation, reversed the effect of ZOL, suggesting that the inhibition of protein(s) geranylgeranylation rather than farnesylation seems to account for ZOL anti-invasive action. To test this hypothesis further, the effect of FTI-277 and GGTI-298 that potently and selectively inhibit FTase and GGTase, respectively, was compared with the action of ZOL on MDA-MB-231 cell invasiveness. These inhibitors have been widely used to identify the prenylated proteins involved in the biological functions of various cell types (Lerner *et al*, 1995; Vogt *et al*, 1996). The incubation of MDA-MB-231 cells with GGTI-298 mimicked the anti-invasive effect of ZOL, whereas FTI-277 was devoid of effect. Thus, inhibition of protein geranylgeranylation seems to be important to explain the anti-invasive action of ZOL. This effect was also mimicked by C3 exoenzyme, which is a specific inhibitor of RhoA, but not for other Rho subfamily members, Rac and Cdc42 (Boquet, 1999). Therefore, it was suggested that the inhibition of cell invasion by ZOL could be related to the inhibition of RhoA cell signalling. This was also supported by our observation showing that ZOL at low concentrations prevents the translocation of RhoA from cytoplasm to the cell membrane. Strikingly, there was a parallel between ZOL inhibition of cell invasion, the decrease in membrane-associated RhoA and the morphological changes characterised by a disorganisation of actin cytoskeleton, as shown by confocal microscopy. Therefore, RhoA inhibition by ZOL could be responsible for its anti-invasive effect on MDA-MB-231 cells by decreasing their motility as a result of the disorganisation of actin cytoskeleton accompanied by a loss of stress fibres. Additionally, Ras inactivation did not appear to be involved in the inhibition of MDA-MB-231 cell invasion because, at the same low concentration, Ras is not inactivated by ZOL, as clearly evidenced by its membrane localisation. Finally, the decrease in cell invasion induced by ZOL was not modified by treatment with 10 *μ*M SQUA, the late metabolite in the cholesterol synthesis pathway, suggesting that regulation of cellular cholesterol level was not involved in this effect.

In contrast to the effect of ZOL on cell invasion, the inhibition of cell proliferation seems independent of RhoA inactivation because at the concentration for which RhoA is inhibited, cell proliferation and apoptosis are unaltered. This inhibition seems more likely due to the inhibition of Ras prenylation as recently demonstrated by Senaratne *et al* (2002).

In the second part of this study, it was shown that ZOL inhibits the chemotactic effect induced by the chemokine SDF-1 on MDA-MB-231 cells. This observation constitutes an important addition to the mechanistic understanding of how BPs, given in adjuvant setting, could prevent the development of bone metastases as shown by two clinical trials (Diel *et al*, 1998; Powles *et al*, 2002). Moreover, we attempted to elucidate whether this effect of ZOL could be explained by the inhibition of the MVA pathway, and more particularly by RhoA inactivation. However, this inhibition was incompletely circumvented by GGOH and only partially mimicked by GGTI-298, suggesting that in contrast to the inhibitory effect induced by ZOL on cell invasion, which could be mainly explained by the disorganisation of cytoskeleton induced by RhoA inhibition, an additional mechanism may occur. Indeed, we demonstrated that the ZOL induces also a potent inhibition of CXCR-4 expression on MDA-MB-231 cells, which was not reversed by GGOH and not mimicked by C3 exoenzyme. Consequently, at least two mechanisms cooperate to induce this inhibiting effect induced by ZOL; one related to defective actin stress fibres formation responsible for the loss of traction forces required for cell motility, which is RhoA-dependent, and the other related to decreased CXCR-4 expression, which is RhoA-independent. This effect of ZOL could be crucial for the inhibition of cancer cell metastasis in bone, as it was reported that neutralising anti-human CXCR-4 monoclonal antibody suppresses metastases in a breast-cancer model (Müller *et al*, 2001).

Finally, in bone, breast-cancer cells interact mainly with bone-resorbing osteoclasts, supplying them with stimulatory factors, which lead to disruption of bone structure and release of stroma-bound factors that in turn stimulate the growth of cancer cells (Yin *et al*, 1999). Invasive breast cancers, which constitutively express inducible Cox-2, enhance osteoclast formation through the production of high levels of PGE2 and subsequently an increase of osteolysis (Ono *et al*, 2002). As an important mechanism involved in the effect of BPs in bone marrow metastasis is the reduction of osteoclast-induced bone osteolysis, we also analysed whether ZOL could inhibit osteoclast activation by cancer cells. In this study, it was demonstrated that Cox-2 mRNA synthesis is greatly reduced by 1 *μ*M ZOL treatment for 6 h. This was responsible for the decrease in Cox-2 protein level and the consequent decrease in PGE2 secretion by MDA-MB-231 cells. These decreases in Cox-2 expression and PGE2 secretion by ZOL are independent of RhoA inactivation as they were not mimicked by C3 exoenzyme and were not reversed by GGOH. Whereas Ras was reported to be involved in Cox-2 expression in these cancer cells (Gilhooly and Rose, 1999), Ras inhibition does not appear to be involved in this Cox-2 inhibition by ZOL, as it occurs at low concentrations that did not inhibit Ras activation. Although the molecular mechanism of this inhibition was not yet elucidated, it is suggested that it could contribute to the beneficial effect induced by ZOL on the inhibition of metastatic breast-cancer-mediated osteolysis. Interestingly, since ZOL, with its high affinity to hydroxyapatite, is concentrated on the bone surface, its effect could be more important than that induced by other Cox-2 inhibitors, which although reducing breast-cancer metastasis, have a reduced affinity for bone in comparison to ZOL (Kundu and Fulton, 2002).

In summary, our results suggest that ZOL could be used not only to treat cancer metastasis-induced osteolysis but also to prevent metastasis. The growth inhibition of osteoclasts has been suggested as the main mechanism of the inhibitory effect of ZOL on bone metastases. In this study, a new concept was proposed by which, in breast-cancer cells, ZOL inhibits the production of PGE2, suggesting a decreased cooperation between osteoclasts and cancer cells for inducing osteolysis. In addition, ZOL induces a potent inhibition of both breast-cancer cell invasion and SDF-1-mediated chemotactic effect. It may be useful to correlate these results with clinical trials to test the efficacy of ZOL on the prevention of metastases in patients with highly aggressive breast cancer. In addition, as both Cox-2 and CXCR-4 inhibitions are RhoA-independent, further investigations are required to determine whether the invasion of cancer cells without RhoA activation could be reduced by ZOL.
